# Supplementary material for: Identification of photoperiod-induced specific miRNAs in the adrenal glands of Sunite sheep (Ovis aries)
Source: Front Vet Sci. 2022 Jul 22;9:888207. doi: 10.3389/fvets.2022.888207 (PMC9354845; doi:10.3389/fvets.2022.888207)
Supplement: Supplementary file 1 [file Data_Sheet_1.ZIP › Supplementary file/miRDeep2 file.zip!/miRDeep2 file/Novel_Structure/SP42A2/Novel_666.pdf]

Provisional ID	:	NC_019458.2_1668
Score total	:	1.4
Score for star read(s)	:	-1.3
Score for read counts	:	0
Score for mfe	:	1.1
Score for randfold	:	1.6
Score for cons. seed	:	
Total read count	:	4957
Mature read count	:	4957
Loop read count	:	0
Star read count	:	0

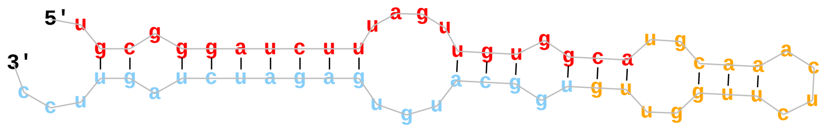

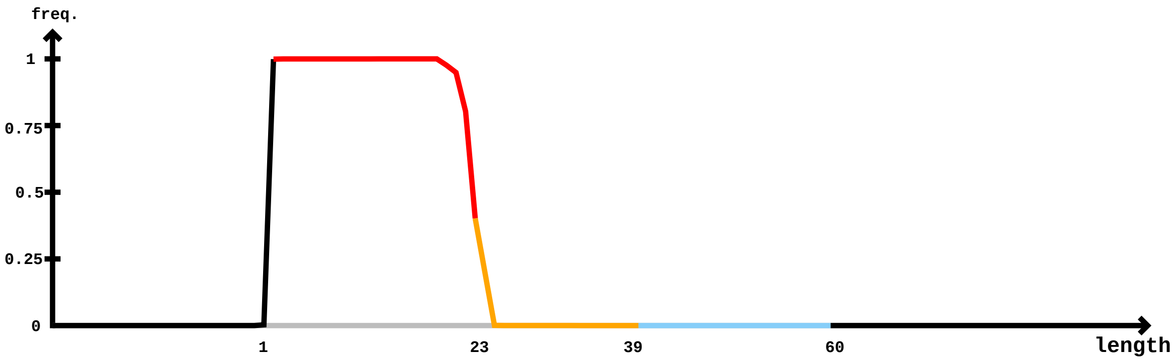

Mature **Star**

```
5'- ucgucagucuuuguuuugggu ugcgggaucuuuaguuguggca ugcaaacucuugguug uggcaugugagaucuaguucc ccaacaagggauggaugcuuacuccuuggaag  -3'   exp
    .((((.((((((...(((((..((.(((((((....(((.(((..(((....)))..))).)))...))))))).))..)))))..)))))).))))...............   reads   mm   sample
    ....................uugcgggaucuuuaguuguggc......................................................................   2       0    seq
    ....................Augcgggaucuuuaguuguggca.....................................................................   12      1    seq
    .....................ugcAggaucuuuaguugu.........................................................................   1       1    seq
    .....................ugUgggaucuuuaguugu.........................................................................   1       1    seq
    .....................ugcgggaucuuuaguugC.........................................................................   12      1    seq
    .....................ugAgggaucuuuaguugu.........................................................................   1       1    seq
    .....................ugcgggaucuuuaguugA.........................................................................   1       1    seq
    .....................ugcgggaucuuuaguugu.........................................................................   100     0    seq
    .....................ugcgAgaucuuuaguugu.........................................................................   1       1    seq
    .....................ugcgggaucuuuaguuguA........................................................................   31      1    seq
    .....................ugcgggaucuuuaguugug........................................................................   105     0    seq
    .....................ugcgggaucuuuaguugugU.......................................................................   34      1    seq
    .....................ugcgggaucuuuUguugugg.......................................................................   1       1    seq
    .....................ugcgggaucuuuaguuguAg.......................................................................   2       1    seq
    .....................ugcgAgaucuuuaguugugg.......................................................................   5       1    seq
    .....................ugcgggaucuuuaguugugA.......................................................................   180     1    seq
    .....................ugcgggaucuuuaguugAgg.......................................................................   1       1    seq
    .....................ugcgggaucuuuaguugugg.......................................................................   497     0    seq
    .....................ugcgggaucuuuaguugCgg.......................................................................   3       1    seq
    .....................ugcgggaucuuuaguuguAgc......................................................................   2       1    seq
    .....................ugcggAaucuuuaguuguggc......................................................................   1       1    seq
    .....................ugcgggaucuuuaguuguggU......................................................................   552     1    seq
    .....................ugcgggaucuuuaguugugUc......................................................................   2       1    seq
    .....................ugcgggaucuuuaguugCggc......................................................................   4       1    seq
    .....................ugcgggaucuuuaguugugAc......................................................................   2       1    seq
    .....................ugUgggaucuuuaguuguggc......................................................................   5       1    seq
    .....................ugcgggaucuuuaguuguggc......................................................................   1418    0    seq
    .....................ugcgggaucuuuaguuguAgca.....................................................................   2       1    seq
    .....................ugcgggaucuuuaguuguggca.....................................................................   1779    0    seq
    .....................ugcgAgaucuuuaguuguggca.....................................................................   4       1    seq
    .....................ugcAggaucuuuaguuguggca.....................................................................   27      1    seq
    .....................ugcgggaucuuuUguuguggca.....................................................................   1       1    seq
    .....................ugcgggaucuuuaguuguggUa.....................................................................   17      1    seq
    .....................ugUgggaucuuuaguuguggca.....................................................................   6       1    seq
```

Mature **Star**

```
ucgucagucuuuguuuuggguugcgggaucuuuaguuguggcaugcaaacucuugguuguggcaugugagaucuaguuccccaacaagggauggaugcuuacuccuuggaag
.....................ugcgggaucuuuagCuguggca.....................................................................    5    1    seq
.....................ugcgggaucuuuaguuguAgcau....................................................................    1    1    seq
.....................ugcgggaucuuuaguuguggcau....................................................................    116  0    seq
.....................ugcAggaucuuuaguuguggcau....................................................................    1    1    seq
.....................ugcgggaucuuuaguuguggcaC....................................................................    15   1    seq
.....................ugcgggaucuuuaguuguggcauC...................................................................    2    1    seq
.....................ugcgggaucuuuaguuguggcauA...................................................................    1    1    seq
......................gcgggaucuuuaguugugg.......................................................................    1    0    seq
......................gUgggaucuuuaguuguggc......................................................................    1    1    seq
......................gcgggaucuuuaguuguggca.....................................................................    2    0    seq
................................uaguuguggcGugcaaacucu...........................................................    1    1    seq
```
